# *N*′-(5-Bromo-2-methoxy­benzyl­idene)-3-hydroxy­benzohydrazide methanol hemisolvate

**DOI:** 10.1107/S1600536808018205

**Published:** 2008-06-19

**Authors:** Zhi Zhou

**Affiliations:** a Department of Chemistry, Kaili College, Kaili Guizhou 556000, People’s Republic of China

## Abstract

The asymmetric unit of the title compound, C_15_H_13_BrN_2_O_3_·0.5CH_3_OH, contains two Schiff base mol­ecules and a methanol mol­ecule of crystallization. The dihedral angles between the benzene rings in the two mol­ecules are 23.8 (2) and 18.6 (2)°. In the crystal structure, mol­ecules are linked through inter­molecular N—H⋯O, O—H⋯O and O—H⋯N hydrogen bonds, forming a three-dimensional network.

## Related literature

For related literature, see: Zhou & Tang (2007 ▶); Zhou & Xiao (2007 ▶). For related structures, see: Ali *et al.* (2007 ▶); Butcher *et al.* (2007 ▶); He (2008 ▶); Jing & Yu (2007 ▶); Nie (2008 ▶).
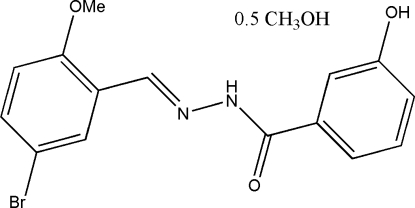


## Experimental

### 

#### Crystal data

C_15_H_13_BrN_2_O_3_·0.5CH_4_O
*M* *_r_* = 365.21
Monoclinic, 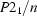

*a* = 12.906 (2) Å
*b* = 11.177 (2) Å
*c* = 22.607 (3) Å
β = 93.706 (3)°
*V* = 3254.3 (9) Å^3^
*Z* = 8
Mo *K*α radiation
μ = 2.54 mm^−1^
*T* = 298 (2) K
0.20 × 0.18 × 0.17 mm

#### Data collection

Bruker SMART CCD area-detector diffractometer
Absorption correction: multi-scan (*SADABS*; Bruker, 2001 ▶) *T*_min_ = 0.630, *T*_max_ = 0.672
21623 measured reflections
6725 independent reflections
2610 reflections with *I* > 2σ(*I*)
*R*_int_ = 0.104

#### Refinement

*R*[*F* ^2^ > 2σ(*F* ^2^)] = 0.061
*wR*(*F* ^2^) = 0.170
*S* = 0.98
6725 reflections
409 parameters
2 restraints
H atoms treated by a mixture of independent and constrained refinement
Δρ_max_ = 0.51 e Å^−3^
Δρ_min_ = −0.47 e Å^−3^

### 

Data collection: *SMART* (Bruker, 2007 ▶); cell refinement: *SAINT* (Bruker, 2007 ▶); data reduction: *SAINT*; program(s) used to solve structure: *SHELXTL* (Sheldrick, 2008 ▶); program(s) used to refine structure: *SHELXTL*; molecular graphics: *SHELXTL*; software used to prepare material for publication: *SHELXTL*.

## Supplementary Material

Crystal structure: contains datablocks global, I. DOI: 10.1107/S1600536808018205/at2575sup1.cif

Structure factors: contains datablocks I. DOI: 10.1107/S1600536808018205/at2575Isup2.hkl

Additional supplementary materials:  crystallographic information; 3D view; checkCIF report

## Figures and Tables

**Table 1:** Hydrogen-bond geometry (Å, °)

*D*—H⋯*A*	*D*—H	H⋯*A*	*D*⋯*A*	*D*—H⋯*A*
N4—H4*A*⋯O2^i^	0.902 (10)	2.045 (18)	2.923 (5)	164 (5)
N2—H2⋯O7^i^	0.894 (10)	1.977 (13)	2.866 (5)	173 (5)
O7—H7⋯O3	0.82	1.96	2.737 (5)	157
O6—H6⋯N1^ii^	0.82	2.48	3.140 (5)	138
O6—H6⋯O2^ii^	0.82	2.06	2.777 (5)	146
O3—H3⋯N3^iii^	0.82	2.64	3.110 (6)	118
O3—H3⋯O5^iii^	0.82	1.92	2.692 (5)	157

Symmetry codes: (i) 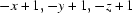; (ii) 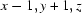; (iii) 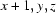.

**Fig. 1.**
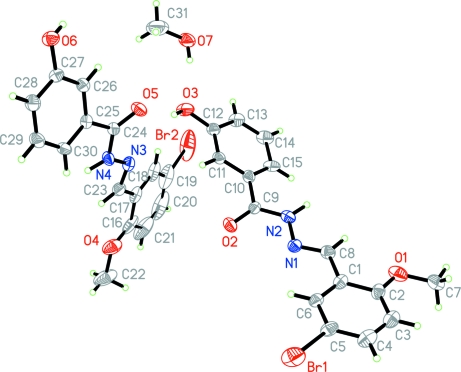
The molecular structure of (I), with displacement ellipsoids drawn at the 30% probability level.

**Fig. 2.**
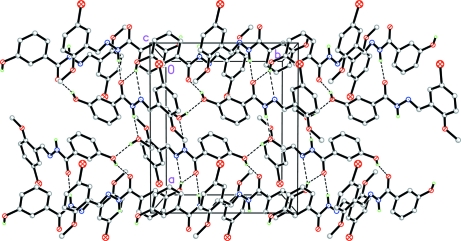
The molecular packing of (I), viewed along the c axis. Hydrogen bonds are shown as dashed lines.

## supplementary crystallographic information

### Comment

Recently, we have reported two metal complexes with Schiff base ligands (Zhou & Tang, 2007; Zhou & Xiao, 2007). We report herein the crystal structure of the title Schiff base compound (I), Fig. 1.

The asymmetric unit of (I) consists of two Schiff base molecules and a methanol molecule of crystallization. The dihedral angles are 23.8 (2) ° and 18.6 (2) °, respectively, between the benzene rings (C1-C6) and (C10-C15) for molecule A, and (C16-C21), (C25-C3015) for molecule B. All the bond values are comparable to the similar compounds (Ali *et al.*, 2007; Nie, 2008; He, 2008; Butcher *et al.*, 2007; Jing & Yu, 2007).

In the crystal structure, molecules are linked through intermolecular N–H···O, O–H···O and O—H···N hydrogen bonds (Table 1) to form a three-dimensional network (Fig. 2).

### Experimental

2-Methoxy-5-bromobenzaldehyde (1.0 mmol, 215.0 mg) and 3-hydroxybenzohydrazide (1.0 mmol, 152.1 mg) were dissolved in methanol (30 ml). The mixture was stirred at reflux for 30 min to give a colourless solution. After keeping the solution in air for a few days, colourless block-like crystals were formed.

### Refinement

H2 and H4A were located in a difference Fourier map and refined isotropically, with *U*_iso_ fixed at 0.08 Å^2^. Other H atoms were positioned geometrically and refined using a riding model with d(O–H) = 0.82 Å, *U*_iso_ = 1.5*U*_eq_(O), and d(C–H) = 0.93 - 0.96 Å, *U*_iso_ = 1.2 or 1.5*U*_eq_(C).

### Crystal data

**Table:**

C_15_H_13_BrN_2_O_3_·0.5CH_4_O	*F*_000_ = 1480
*M**_r_* = 365.21	*D*_x_ = 1.491 Mg m^−^^3^
Monoclinic, *P*2_1_/*n*	Mo *K*α radiation λ = 0.71073 Å
Hall symbol: -P 2yn	Cell parameters from 1690 reflections
*a* = 12.906 (2) Å	θ = 2.4–24.1º
*b* = 11.177 (2) Å	µ = 2.54 mm^−^^1^
*c* = 22.607 (3) Å	*T* = 298 (2) K
β = 93.706 (3)º	Block, colourless
*V* = 3254.3 (9) Å^3^	0.20 × 0.18 × 0.17 mm
*Z* = 8	

### Data collection

**Table:**

Bruker SMART CCD area-detector diffractometer	6725 independent reflections
Radiation source: fine-focus sealed tube	2610 reflections with *I* > 2σ(*I*)
Monochromator: graphite	*R*_int_ = 0.104
*T* = 298(2) K	θ_max_ = 26.5º
ω scans	θ_min_ = 1.8º
Absorption correction: multi-scan(SADABS; Bruker, 2001)	*h* = −16→16
*T*_min_ = 0.630, *T*_max_ = 0.672	*k* = −13→14
21623 measured reflections	*l* = −28→27

### Refinement

**Table:**

Refinement on *F*^2^	Secondary atom site location: difference Fourier map
Least-squares matrix: full	Hydrogen site location: inferred from neighbouring sites
*R*[*F*^2^ > 2σ(*F*^2^)] = 0.061	H atoms treated by a mixture of independent and constrained refinement
*wR*(*F*^2^) = 0.170	*w* = 1/[σ^2^(*F*_o_^2^) + (0.0522*P*)^2^] where *P* = (*F*_o_^2^ + 2*F*_c_^2^)/3
*S* = 0.98	(Δ/σ)_max_ = 0.001
6725 reflections	Δρ_max_ = 0.51 e Å^−^^3^
409 parameters	Δρ_min_ = −0.47 e Å^−^^3^
2 restraints	Extinction correction: none
Primary atom site location: structure-invariant direct methods	

### Special details

**Table:**

Geometry. All e.s.d.'s (except the e.s.d. in the dihedral angle between two l.s. planes) are estimated using the full covariance matrix. The cell e.s.d.'s are taken into account individually in the estimation of e.s.d.'s in distances, angles and torsion angles; correlations between e.s.d.'s in cell parameters are only used when they are defined by crystal symmetry. An approximate (isotropic) treatment of cell e.s.d.'s is used for estimating e.s.d.'s involving l.s. planes.
Refinement. Refinement of *F*^2^ against ALL reflections. The weighted *R*-factor *wR* and goodness of fit *S* are based on *F*^2^, conventional *R*-factors *R* are based on *F*, with *F* set to zero for negative *F*^2^. The threshold expression of *F*^2^ > σ(*F*^2^) is used only for calculating *R*-factors(gt) *etc*. and is not relevant to the choice of reflections for refinement. *R*-factors based on *F*^2^ are statistically about twice as large as those based on *F*, and *R*- factors based on ALL data will be even larger.

### Fractional atomic coordinates and isotropic or equivalent isotropic displacement parameters (Å^2^)

**Table:**

	*x*	*y*	*z*	*U*_iso_*/*U*_eq_	
Br1	0.89733 (6)	−0.03746 (6)	0.19990 (3)	0.0896 (3)	
Br2	−0.27606 (8)	0.47236 (11)	0.74505 (4)	0.1632 (5)	
O1	0.4978 (3)	−0.1249 (3)	0.31384 (17)	0.0705 (11)	
O2	0.8013 (2)	0.2809 (3)	0.44744 (14)	0.0499 (9)	
O3	0.6978 (3)	0.6557 (3)	0.55948 (18)	0.0680 (11)	
H3	0.7389	0.6728	0.5346	0.102*	
O4	0.1231 (4)	0.3770 (4)	0.6320 (2)	0.0868 (14)	
O5	−0.1758 (3)	0.7734 (3)	0.48927 (15)	0.0563 (10)	
O6	−0.0715 (3)	1.1472 (3)	0.37821 (15)	0.0551 (10)	
H6	−0.1226	1.1603	0.3970	0.083*	
O7	0.5718 (3)	0.8519 (4)	0.5649 (3)	0.0984 (16)	
H7	0.6018	0.7923	0.5537	0.148*	
N1	0.6877 (3)	0.1219 (3)	0.38704 (17)	0.0435 (10)	
N2	0.6465 (3)	0.1906 (4)	0.43049 (18)	0.0454 (11)	
N3	−0.0639 (3)	0.6176 (4)	0.55665 (18)	0.0489 (11)	
N4	−0.0209 (3)	0.6887 (4)	0.51548 (18)	0.0448 (11)	
C1	0.6526 (4)	−0.0203 (5)	0.3091 (2)	0.0476 (14)	
C2	0.5840 (4)	−0.1083 (5)	0.2848 (2)	0.0494 (14)	
C3	0.6097 (5)	−0.1693 (5)	0.2347 (2)	0.0599 (16)	
H3A	0.5641	−0.2261	0.2179	0.072*	
C4	0.7018 (5)	−0.1475 (5)	0.2094 (2)	0.0623 (16)	
H4	0.7181	−0.1892	0.1756	0.075*	
C5	0.7690 (4)	−0.0647 (5)	0.2338 (2)	0.0532 (15)	
C6	0.7453 (4)	−0.0001 (4)	0.2831 (2)	0.0466 (14)	
H6A	0.7916	0.0571	0.2990	0.056*	
C7	0.4258 (4)	−0.2131 (5)	0.2916 (3)	0.0795 (19)	
H7A	0.4041	−0.1949	0.2512	0.119*	
H7B	0.3664	−0.2133	0.3151	0.119*	
H7C	0.4582	−0.2904	0.2936	0.119*	
C8	0.6233 (4)	0.0517 (5)	0.3595 (2)	0.0485 (14)	
H8	0.5560	0.0460	0.3717	0.058*	
C9	0.7095 (4)	0.2717 (4)	0.4583 (2)	0.0403 (12)	
C10	0.6619 (4)	0.3512 (4)	0.5019 (2)	0.0424 (13)	
C11	0.7043 (4)	0.4646 (4)	0.5105 (2)	0.0441 (13)	
H11	0.7613	0.4877	0.4901	0.053*	
C12	0.6614 (4)	0.5429 (5)	0.5494 (2)	0.0475 (13)	
C13	0.5772 (4)	0.5085 (5)	0.5794 (2)	0.0562 (15)	
H13	0.5487	0.5610	0.6058	0.067*	
C14	0.5352 (4)	0.3978 (5)	0.5707 (2)	0.0610 (16)	
H14	0.4775	0.3758	0.5908	0.073*	
C15	0.5772 (4)	0.3173 (5)	0.5321 (2)	0.0533 (15)	
H15	0.5486	0.2414	0.5267	0.064*	
C16	0.0358 (7)	0.3912 (5)	0.6599 (3)	0.075 (2)	
C17	−0.0323 (5)	0.4802 (5)	0.6358 (2)	0.0563 (16)	
C18	−0.1248 (5)	0.5010 (5)	0.6608 (3)	0.0716 (19)	
H18	−0.1693	0.5598	0.6448	0.086*	
C19	−0.1527 (7)	0.4368 (8)	0.7089 (3)	0.105 (3)	
C20	−0.0841 (10)	0.3495 (9)	0.7327 (4)	0.134 (5)	
H20	−0.1013	0.3062	0.7658	0.161*	
C21	0.0056 (9)	0.3278 (7)	0.7085 (3)	0.116 (4)	
H21	0.0491	0.2684	0.7247	0.140*	
C22	0.1976 (6)	0.2885 (6)	0.6549 (3)	0.125 (3)	
H22A	0.2229	0.3105	0.6942	0.187*	
H22B	0.2546	0.2846	0.6297	0.187*	
H22C	0.1645	0.2116	0.6559	0.187*	
C23	0.0025 (4)	0.5525 (4)	0.5873 (2)	0.0501 (14)	
H23	0.0719	0.5518	0.5784	0.060*	
C24	−0.0810 (4)	0.7700 (4)	0.4857 (2)	0.0422 (13)	
C25	−0.0264 (4)	0.8531 (4)	0.4470 (2)	0.0411 (13)	
C26	−0.0762 (4)	0.9601 (4)	0.43118 (19)	0.0412 (12)	
H26	−0.1412	0.9771	0.4445	0.049*	
C27	−0.0283 (4)	1.0406 (5)	0.3956 (2)	0.0429 (13)	
C28	0.0680 (4)	1.0164 (5)	0.3763 (2)	0.0561 (15)	
H28	0.1007	1.0720	0.3532	0.067*	
C29	0.1162 (4)	0.9099 (5)	0.3910 (2)	0.0577 (15)	
H29	0.1806	0.8927	0.3768	0.069*	
C30	0.0696 (4)	0.8282 (4)	0.4267 (2)	0.0455 (13)	
H30	0.1030	0.7568	0.4370	0.055*	
C31	0.6289 (6)	0.9488 (7)	0.5556 (5)	0.173 (5)	
H31A	0.5889	1.0194	0.5622	0.260*	
H31B	0.6491	0.9484	0.5154	0.260*	
H31C	0.6898	0.9483	0.5823	0.260*	
H2	0.5782 (11)	0.183 (5)	0.434 (2)	0.080*	
H4A	0.0489 (9)	0.695 (5)	0.520 (2)	0.080*	

### Atomic displacement parameters (Å^2^)

**Table:**

	*U*^11^	*U*^22^	*U*^33^	*U*^12^	*U*^13^	*U*^23^
Br1	0.1071 (6)	0.0787 (5)	0.0892 (5)	0.0026 (4)	0.0552 (4)	0.0009 (4)
Br2	0.1725 (10)	0.2271 (12)	0.0980 (7)	−0.1320 (9)	0.0695 (6)	−0.0473 (7)
O1	0.057 (3)	0.071 (3)	0.084 (3)	−0.018 (2)	0.008 (2)	−0.022 (2)
O2	0.035 (2)	0.056 (2)	0.059 (2)	0.0003 (18)	0.0038 (17)	−0.0118 (18)
O3	0.067 (3)	0.046 (2)	0.096 (3)	−0.010 (2)	0.042 (2)	−0.018 (2)
O4	0.119 (4)	0.055 (3)	0.082 (3)	0.019 (3)	−0.028 (3)	0.003 (2)
O5	0.040 (2)	0.060 (2)	0.070 (3)	0.0084 (19)	0.0117 (19)	0.0173 (19)
O6	0.061 (3)	0.046 (2)	0.060 (2)	0.0045 (19)	0.0187 (19)	0.0093 (19)
O7	0.047 (3)	0.057 (3)	0.193 (5)	−0.005 (2)	0.028 (3)	−0.036 (3)
N1	0.047 (3)	0.036 (3)	0.048 (3)	0.000 (2)	0.004 (2)	−0.008 (2)
N2	0.039 (3)	0.044 (3)	0.054 (3)	−0.006 (2)	0.012 (2)	−0.017 (2)
N3	0.049 (3)	0.046 (3)	0.052 (3)	−0.010 (2)	0.005 (2)	−0.001 (2)
N4	0.033 (3)	0.048 (3)	0.054 (3)	−0.004 (2)	0.004 (2)	0.011 (2)
C1	0.060 (4)	0.041 (3)	0.042 (3)	0.002 (3)	0.000 (3)	0.001 (3)
C2	0.055 (4)	0.041 (3)	0.051 (4)	0.007 (3)	−0.004 (3)	−0.006 (3)
C3	0.081 (5)	0.045 (4)	0.051 (4)	0.001 (3)	−0.011 (3)	−0.006 (3)
C4	0.094 (5)	0.046 (4)	0.047 (4)	0.002 (4)	0.012 (3)	−0.009 (3)
C5	0.073 (4)	0.043 (4)	0.046 (3)	0.000 (3)	0.019 (3)	0.005 (3)
C6	0.054 (4)	0.036 (3)	0.050 (3)	−0.002 (3)	0.002 (3)	0.002 (3)
C7	0.056 (4)	0.071 (4)	0.110 (5)	−0.014 (4)	−0.009 (4)	0.000 (4)
C8	0.045 (3)	0.049 (4)	0.053 (3)	−0.001 (3)	0.014 (3)	−0.004 (3)
C9	0.041 (3)	0.036 (3)	0.044 (3)	0.002 (3)	0.004 (3)	0.001 (3)
C10	0.038 (3)	0.042 (3)	0.047 (3)	0.002 (3)	0.004 (2)	−0.005 (3)
C11	0.034 (3)	0.045 (3)	0.054 (3)	0.000 (3)	0.011 (2)	−0.004 (3)
C12	0.042 (3)	0.043 (3)	0.059 (3)	−0.002 (3)	0.015 (3)	−0.007 (3)
C13	0.064 (4)	0.050 (4)	0.058 (4)	−0.003 (3)	0.025 (3)	−0.018 (3)
C14	0.064 (4)	0.065 (4)	0.057 (4)	−0.011 (3)	0.028 (3)	−0.013 (3)
C15	0.059 (4)	0.049 (4)	0.053 (4)	−0.020 (3)	0.013 (3)	−0.011 (3)
C16	0.134 (7)	0.033 (4)	0.055 (5)	−0.025 (4)	−0.021 (5)	0.008 (3)
C17	0.075 (4)	0.049 (4)	0.044 (4)	−0.025 (3)	−0.002 (3)	0.003 (3)
C18	0.096 (5)	0.069 (5)	0.048 (4)	−0.043 (4)	−0.002 (4)	−0.001 (3)
C19	0.151 (8)	0.114 (7)	0.050 (5)	−0.074 (6)	0.016 (5)	−0.008 (5)
C20	0.235 (15)	0.115 (9)	0.052 (6)	−0.108 (10)	0.002 (7)	0.018 (5)
C21	0.219 (12)	0.061 (5)	0.063 (6)	−0.039 (7)	−0.034 (6)	0.020 (5)
C22	0.175 (8)	0.067 (5)	0.120 (6)	0.046 (5)	−0.082 (6)	−0.020 (4)
C23	0.054 (4)	0.042 (3)	0.054 (4)	−0.001 (3)	0.002 (3)	0.001 (3)
C24	0.038 (3)	0.041 (3)	0.047 (3)	−0.001 (3)	0.001 (3)	−0.004 (3)
C25	0.040 (3)	0.042 (3)	0.041 (3)	−0.006 (3)	0.002 (2)	−0.001 (3)
C26	0.040 (3)	0.048 (3)	0.035 (3)	0.000 (3)	0.000 (2)	−0.003 (3)
C27	0.050 (3)	0.038 (3)	0.042 (3)	0.002 (3)	0.005 (3)	−0.002 (3)
C28	0.051 (4)	0.057 (4)	0.062 (4)	−0.004 (3)	0.018 (3)	0.010 (3)
C29	0.043 (3)	0.061 (4)	0.071 (4)	0.003 (3)	0.022 (3)	0.005 (3)
C30	0.038 (3)	0.046 (3)	0.052 (3)	0.009 (3)	0.005 (3)	0.000 (3)
C31	0.093 (6)	0.077 (6)	0.357 (15)	−0.022 (5)	0.076 (8)	−0.013 (8)

### Geometric parameters (Å, °)

**Table:**

Br1—C5	1.893 (5)	C10—C11	1.389 (6)
Br2—C19	1.879 (9)	C11—C12	1.382 (6)
O1—C2	1.342 (6)	C11—H11	0.9300
O1—C7	1.423 (6)	C12—C13	1.372 (7)
O2—C9	1.230 (5)	C13—C14	1.360 (7)
O3—C12	1.360 (5)	C13—H13	0.9300
O3—H3	0.8200	C14—C15	1.387 (7)
O4—C16	1.336 (8)	C14—H14	0.9300
O4—C22	1.452 (6)	C15—H15	0.9300
O5—C24	1.233 (5)	C16—C21	1.384 (9)
O6—C27	1.363 (5)	C16—C17	1.413 (8)
O6—H6	0.8200	C17—C18	1.374 (8)
O7—C31	1.335 (7)	C17—C23	1.456 (7)
O7—H7	0.8200	C18—C19	1.370 (9)
N1—C8	1.276 (5)	C18—H18	0.9300
N1—N2	1.379 (5)	C19—C20	1.401 (12)
N2—C9	1.346 (6)	C20—C21	1.335 (12)
N2—H2	0.894 (10)	C20—H20	0.9300
N3—C23	1.290 (6)	C21—H21	0.9300
N3—N4	1.369 (5)	C22—H22A	0.9600
N4—C24	1.347 (6)	C22—H22B	0.9600
N4—H4A	0.902 (10)	C22—H22C	0.9600
C1—C6	1.384 (7)	C23—H23	0.9300
C1—C2	1.411 (7)	C24—C25	1.484 (6)
C1—C8	1.465 (7)	C25—C30	1.377 (6)
C2—C3	1.380 (7)	C25—C26	1.393 (6)
C3—C4	1.374 (7)	C26—C27	1.380 (6)
C3—H3A	0.9300	C26—H26	0.9300
C4—C5	1.361 (7)	C27—C28	1.371 (7)
C4—H4	0.9300	C28—C29	1.374 (7)
C5—C6	1.380 (7)	C28—H28	0.9300
C6—H6A	0.9300	C29—C30	1.382 (6)
C7—H7A	0.9600	C29—H29	0.9300
C7—H7B	0.9600	C30—H30	0.9300
C7—H7C	0.9600	C31—H31A	0.9600
C8—H8	0.9300	C31—H31B	0.9600
C9—C10	1.489 (6)	C31—H31C	0.9600
C10—C15	1.379 (6)		
			
C2—O1—C7	117.8 (4)	C10—C15—C14	119.2 (5)
C12—O3—H3	109.5	C10—C15—H15	120.4
C16—O4—C22	118.1 (6)	C14—C15—H15	120.4
C27—O6—H6	109.5	O4—C16—C21	127.1 (8)
C31—O7—H7	109.5	O4—C16—C17	115.0 (6)
C8—N1—N2	114.9 (4)	C21—C16—C17	117.8 (8)
C9—N2—N1	117.3 (4)	C18—C17—C16	119.7 (6)
C9—N2—H2	126 (3)	C18—C17—C23	122.3 (6)
N1—N2—H2	116 (3)	C16—C17—C23	117.9 (6)
C23—N3—N4	114.1 (4)	C19—C18—C17	121.2 (7)
C24—N4—N3	119.1 (4)	C19—C18—H18	119.4
C24—N4—H4A	123 (3)	C17—C18—H18	119.4
N3—N4—H4A	115 (3)	C18—C19—C20	118.5 (9)
C6—C1—C2	119.1 (5)	C18—C19—Br2	120.6 (8)
C6—C1—C8	120.9 (5)	C20—C19—Br2	120.8 (7)
C2—C1—C8	119.9 (5)	C21—C20—C19	120.8 (9)
O1—C2—C3	125.8 (5)	C21—C20—H20	119.6
O1—C2—C1	115.2 (5)	C19—C20—H20	119.6
C3—C2—C1	119.1 (5)	C20—C21—C16	121.9 (10)
C4—C3—C2	120.9 (5)	C20—C21—H21	119.0
C4—C3—H3A	119.5	C16—C21—H21	119.0
C2—C3—H3A	119.5	O4—C22—H22A	109.5
C5—C4—C3	119.9 (5)	O4—C22—H22B	109.5
C5—C4—H4	120.1	H22A—C22—H22B	109.5
C3—C4—H4	120.1	O4—C22—H22C	109.5
C4—C5—C6	121.0 (5)	H22A—C22—H22C	109.5
C4—C5—Br1	119.8 (4)	H22B—C22—H22C	109.5
C6—C5—Br1	119.2 (4)	N3—C23—C17	119.4 (5)
C5—C6—C1	120.0 (5)	N3—C23—H23	120.3
C5—C6—H6A	120.0	C17—C23—H23	120.3
C1—C6—H6A	120.0	O5—C24—N4	122.0 (5)
O1—C7—H7A	109.5	O5—C24—C25	122.1 (5)
O1—C7—H7B	109.5	N4—C24—C25	116.0 (5)
H7A—C7—H7B	109.5	C30—C25—C26	119.9 (5)
O1—C7—H7C	109.5	C30—C25—C24	122.8 (5)
H7A—C7—H7C	109.5	C26—C25—C24	117.4 (4)
H7B—C7—H7C	109.5	C27—C26—C25	119.6 (5)
N1—C8—C1	121.7 (5)	C27—C26—H26	120.2
N1—C8—H8	119.2	C25—C26—H26	120.2
C1—C8—H8	119.2	O6—C27—C28	116.3 (5)
O2—C9—N2	121.6 (5)	O6—C27—C26	123.3 (5)
O2—C9—C10	121.7 (5)	C28—C27—C26	120.4 (5)
N2—C9—C10	116.7 (4)	C27—C28—C29	120.0 (5)
C15—C10—C11	119.9 (4)	C27—C28—H28	120.0
C15—C10—C9	122.5 (5)	C29—C28—H28	120.0
C11—C10—C9	117.5 (4)	C28—C29—C30	120.4 (5)
C12—C11—C10	119.8 (5)	C28—C29—H29	119.8
C12—C11—H11	120.1	C30—C29—H29	119.8
C10—C11—H11	120.1	C25—C30—C29	119.7 (5)
O3—C12—C13	117.0 (5)	C25—C30—H30	120.1
O3—C12—C11	123.1 (5)	C29—C30—H30	120.1
C13—C12—C11	119.9 (5)	O7—C31—H31A	109.5
C14—C13—C12	120.3 (5)	O7—C31—H31B	109.5
C14—C13—H13	119.8	H31A—C31—H31B	109.5
C12—C13—H13	119.8	O7—C31—H31C	109.5
C13—C14—C15	120.8 (5)	H31A—C31—H31C	109.5
C13—C14—H14	119.6	H31B—C31—H31C	109.5
C15—C14—H14	119.6		

### Hydrogen-bond geometry (Å, °)

**Table:**

*D*—H···*A*	*D*—H	H···*A*	*D*···*A*	*D*—H···*A*
N4—H4A···O2^i^	0.902 (10)	2.045 (18)	2.923 (5)	164 (5)
N2—H2···O7^i^	0.894 (10)	1.977 (13)	2.866 (5)	173 (5)
O7—H7···O3	0.82	1.96	2.737 (5)	157
O6—H6···N1^ii^	0.82	2.48	3.140 (5)	138
O6—H6···O2^ii^	0.82	2.06	2.777 (5)	146
O3—H3···N3^iii^	0.82	2.64	3.110 (6)	118
O3—H3···O5^iii^	0.82	1.92	2.692 (5)	157

Symmetry codes: (i) −*x*+1, −*y*+1, −*z*+1; (ii) *x*−1, *y*+1, *z*; (iii) *x*+1, *y*, *z*.
